# Haemodynamic and renal effects of clonidine in an ovine model of severe sepsis and septic acute kidney injury

**DOI:** 10.1186/cc11721

**Published:** 2012-11-14

**Authors:** P Calzavacca, K Ishikawa, K Lu, R Bellomo, CN May

**Affiliations:** 1 Ospedale Uboldo, Cernusco sul Naviglio, Italy; 2 Iwate Medical University, Morioka, Japan; 3 Melbourne university, Florey Neuroscience Institutes, Parkville, Australia; 4 Austin Hospital, Heidelberg, Australia

## Background

In sepsis, the sympathetic nerve activity (SNA) is differentially increased to individual organs [1]. It has been suggested that inhibition of central sympathetic outflow with clonidine would improve outcome in sepsis [2] but the cardiovascular and renal effects of clonidine in sepsis are unknown. Accordingly, we sought to assess the effect of the central α_2_-agonist clonidine on renal function in an ovine model of severe sepsis.

## Methods

Animals had renal and cardiac flow probes implanted to continuously measure the cardiac index (CI) and renal blood flow (RBF). Mean arterial pressure (MAP) was continuously monitored and hourly urine collection was performed. After the first 24 hours of sepsis every animal was randomly and blindly allocated to receive vehicle or clonidine 0.25 μg/kg/hour for 8 hours. Animals were followed for further 40 hours during recovery and, at least 2 weeks later, crossed over to the other arm of the study. Two-way repeated-measures ANOVA was performed and *P *< 0.05 was considered significant. Data are mean ± standard error of the average of 4 hours preceding each time point.

## Results

**Figure 1:**
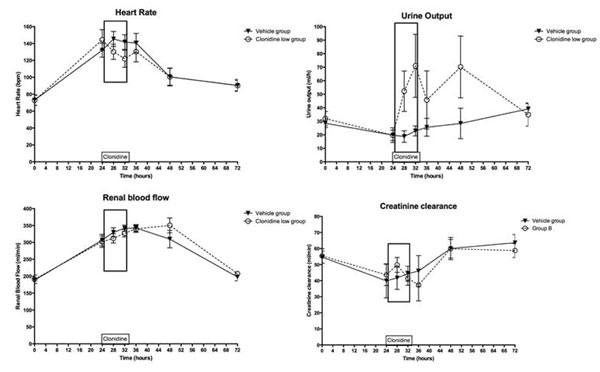
**Haemodynamic and renal effects of clonidine in sepsis**. Data are mean ± standard error. Rectangle: 8 hours of clonidine infusion.

Eight animals per group were studied. Two animals per group died during the first experiment, leaving six animals for crossover. Values presented refer to all eight animals. Animals in both groups had similar baseline values and developed a similar hyperdynamic state with 50% increase in CI, 15 mmHg drop in MAP and threefold increase in arterial lactate. Clonidine (Figure 1) reduced the HR by 23(7) bpm (*P *= 0.013) but did not affect MAP, RBF and lactate levels. Clonidine increased urine output from 23(4) to 71(23) ml/hour (*P *= 0.004), but did not improve creatinine clearance (from 44(6) to 42(4) ml/minute).

## Conclusion

In experimental hyperdynamic sepsis, treatment with clonidine appears safe, but it does not improve the glomerular filtration rate.

## References

[B1] Ramchandra R Wan L Hood SG Frithiof R Bellomo R May CN Septic shock induces distinct changes in sympathetic nerve activity to the heart and kidney in conscious sheep Am J Physiol Regul Integr Comp Physiol 2009 297 R1247 R1253 10.1152/ajpregu.00437.2009 19726712

[B2] Pichot C Geloen A Ghignone M Quintin L Alpha-2 agonists to reduce vasopressor requirements in septic shock? Medical Hypotheses 2010 75 652 656 10.1016/j.mehy.2010.08.010 20817367

